# Climate Change and the Evolution and Fate of the Tangier Islands of Chesapeake Bay, USA

**DOI:** 10.1038/srep17890

**Published:** 2015-12-10

**Authors:** David M. Schulte, Karin M. Dridge, Mark H. Hudgins

**Affiliations:** 1 US Army Corps of Engineers, Norfolk District

## Abstract

Climate change and associated sea level rise (SLR) are already impacting low-lying coastal areas, including islands, throughout the world. Many of these areas are inhabited, many will need to be abandoned in coming decades as SLR continues. We examine the evolution (1850-2013) of the last inhabited offshore island in Virginia waters of Chesapeake Bay USA, the Tangier Islands. Three SLR scenarios, a low, mid, and high, were considered. Since 1850, 66.75% of the islands landmass has been lost. Under the mid-range SLR scenario, much of the remaining landmass is expected to be lost in the next 50 years and the Town will likely need to be abandoned. The high SLR scenario will accelerate the land loss and subsidence, such that the Town may need to be abandoned in as few as 25 years. We propose a conceptual plan that would significantly extend the lifespan of the islands and Town.

The Intergovernmental Panel on Climate Change (IPCC Fifth Assessment Report (AR5)) noted global mean sea level rose significantly since the Industrial Revolution and has been accelerating^1^. The inhabited Tangier Islands, the focus of the present study, lie within a “hot spot” of RSLR (relative sea level rise) in Chesapeake Bay waters bordering the mid-Atlantic (Virginia, USA) significantly higher than the global mean SLR (sea level rise)^2^,^3^,^4^,^5^,^6^,^7^. The Islands, comprised mostly of estuarine wetlands with a number of sandy upland ridges, were settled in the 1700s by European colonists^8^ and have been inhabited since, with 727 people living on the island as of 2013. Using geo-referenced maps dating from 1850–2013, we show the Islands have lost the majority of their landmass since the first accurate map was made in 1850; by 2013, only 33.25% of the 1850 landmass remained. Modeling using ARCGIS and the USGS Digital Shoreline Analysis System showed that, under a mid-range RSLR scenario, most of the remaining landmass of the Islands will be lost within 100 years, and the Islands will likely have to be abandoned in approximately 50 years.

Analysis in the AR5 report lists the following: accelerating rates of global mean SLR: 1.7 mm yr^−1^ (1901–2010), 2.0 mm yr^−1^ (1971–2010), and 3.2 mm yr^−1^ (1993–2010) and predicts 60–100 cm SLR by 2100^1^. Locally, RSLR (relative sea level rise) has been higher, due to a SLR “hot spot” along the Atlantic Coast of North America^2^, part of a region in the North Atlantic above global mean SLR^2^,^3^. This “hot spot” extends from Cape Hatteras, NC to Boston, MA and rose 3.80 ± 1.06 mm yr^−1^ (1970–2009). The Southern Chesapeake Bay region, within this “hot spot,” had a higher rate of SLR (4.40 ± 0.086 mm yr^−1^ from 1955–2007), due to land subsidence (2.101 mm yr^−1^) due to glacial rebound caused by the Laurentide ice sheet melting^4^,^5^, groundwater extraction, as well as after effects of a meteor impact near Cape Charles, VA approximately 35.5 million years ago^6^, which is still causing local land subsidence^7^. Glacial rebound is causing a significant portion of local land subsidence, with groundwater pumping being the other major factor. Aquifer-system compaction is causing more than half of local land subsidence, especially near population and agricultural centers. This compaction can account for more than 50% of observed land subsidence rates in the region and even more so near regions where extensive groundwater pumping is occurring, such as near the cities of West Point, Franklin and Norfolk, Virginia^8^,^9^. Due to the isolation of Tangier Island from major population and agricultural centers responsible for extensive groundwater pumping, groundwater pumping related subsidence at Tangier Island may be less than 50% of total subsidence reported locally, but it is likely significant.

Chesapeake Bay is the largest tidal estuary in USA. It is located along the East Coast of the USA in the mid-Atlantic region, with the northern portion (approximately half) lying within the State of Maryland’s border and the southern portion lying in the State of Virginia’s border. At initial European settlement (1600s), the Chesapeake Bay had hundreds of islands (1-800 HA in size). Many islands (>500) have been lost to SLR and erosion since the 1600s and losses continue^10^. Few islands were ever inhabited (~39), as most lacked sufficient uplands for habitation. Declines in upland areas resulted in abandonment of some of the largest islands in the early 1900s, including Bloodworth^11^, Holland^12^ and Uppards Island^10^. Uppards is one of the Tangier Islands, the focus of the present study.

The Tangier Islands are a complex of what are now several islands, once connected, located in Virginia waters of Chesapeake Bay (Fig. 1) near the border between Virginia and Maryland approximately 151 km southeast of Washington DC. The main remaining Tangier Islands are, from north to south, Goose, Uppards, Port Isobel and Tangier Island. The Tangier Islands lie 22 km east of mainland Virginia and 16.2 km west from the Eastern Shore, a peninsula that partially encloses Chesapeake Bay. The West side of the Bay has deeper waters than those East of the Islands. Due to the longer fetch, wave action along western shores of the Islands is more powerful. Large SAV (seagrass) beds (μ = 296 HA from 2003–13^13^) lie immediately east of the Islands, which protect the SAV from the western fetch. A 1.74-km long rock revetment was constructed along the majority of western shore of Tangier Island in 1989 at a cost of 10.614 million (inflation-adjusted 2014 USD). It has successfully protected a small airport (48.98 HA, 1.5 m above PMSL (Present Mean Sea Level) and nearby Town of Tangier (33.59 HA, 1.46 m above PMSL). The Town is on three upland sand ridges ranging in length from 518–1859 m and up to 396 m wide^14^. It is primarily an isolated fishing community, with a significant portion (13%, the highest total for any town in Chesapeake Bay) of the most valuable fishery in the Bay, blue crabs, *Callinectes sapidus*. This fishery (~30 million USD yr^−1^ ex-vessel revenue in Virginia)^15^ provides the majority of the income for Tangier fishermen. The Town relies on artesian wells drilled approximately 300 m deep into an Eocene era aquifer beneath the Bay, and the average amount of water pumped has been 380 m^3^ day^−1^ (Mills *et al.* 2005), which, when compared to the pumping near large population centers in southeast Virginia that have caused local subsidence near the pumping areas the amount of water pumping by the citizens of the Town of Tangier appears to be insignificant with respect to local subsidence. The construction of a navigation channel between Tangier and Uppards Islands in 1967 may have accelerated local erosion along Uppards due to boat wakes. Placement of dredged material in several landward cells (<10 HA) in the mid-to-late 1900s increased elevation in a few areas, particularly on Port Isobel. Along the eastern shore of Port Isobel, six stone breakwaters were placed in the 2000s for shoreline protection.

Wetlands have significant value, providing ecological services estimated as high as $ 10,000 USD ha^−1^ yr^−1^ (in 2014 USD)^16^. The SAV beds found locally consist of two species, eelgrass (*Zostera marina*) and widgeongrass (*Ruppia maritima*). SAV has both ecological and economic value, providing a wide suite of ecosystem services including erosion control, coastal protection, water quality improvement, nursery habitat for fish and shellfish, particularly blue crabs (MEPS Ralph), and carbon sequestration^17^. SAV has an overall value estimated at over $ 20,000 USD HA^−1^ yr^−1^ 18. The islands provide nesting habitat for waterbirds in Chesapeake Bay, requiring the absence of large terrestrial predators. Bird use has declined as the islands have eroded and lost elevation^19^.

A prior study focused on Uppards Island predicted that Uppards would be completely lost by 2100^20^. In this study, the effects of sea level rise (assumed to continue at present, at the time of the study, rates, which the authors noted was likely conservative), wave fetch, wind speed and direction were examined and the resultant erosion rate was estimated for the Western and Eastern shore of Uppands, Port Isobel and Tangier Island by selecting 10 points along the western and eastern shoreline of all the islands. The study then projected the fate of the islands in 2100. At this time, based on erosion rates extrapolated to 2100, the study authors predicted that Uppards Island would be completely eroded, with relatively small changes occurring to Port Isobel and Tangier Island. No predictions were made as to the future habitability of the Town of Tangier at 2100 or the types of habitat (upland or wetland) that might be present, though study authors did note that interior water had increased over time and that present upland ridges may need to be raised in the future. Our hypotheses are that Uppards and Goose Island will be lost before 2100, and that, without human intervention, the Town of Tangier will have to be abandoned prior to 2100.

## Methods

**Figure 1:**
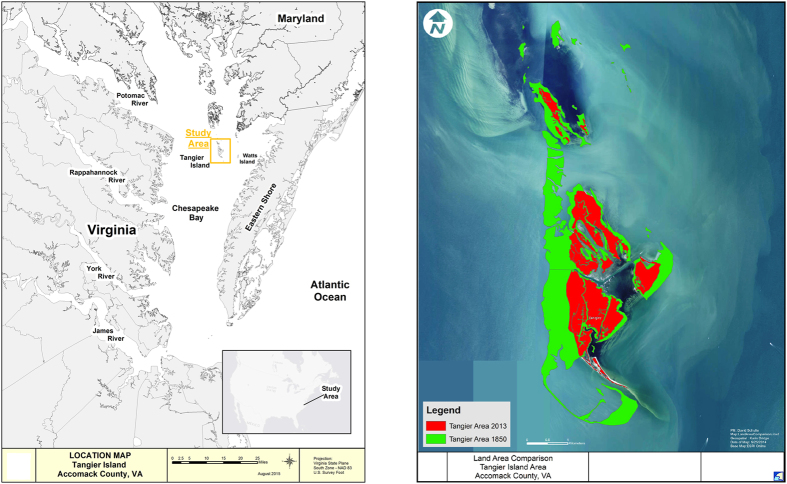
Location and configuration of the Tangier Islands, in Virginia waters of Chesapeake Bay, USA. Map created using ESRI ArcGIS, ArcMap 10.1. Source: Esri, DigitalGlobe, GeoEye, Earthstar Geographics, CNES/Airbus DS, USDA, AEX, Getmapping, Aerogrid, IGN, IGP, swisstopo, and the GIS User Community. For more information on this map, including the terms of use, visit: http://goto.arcgisonline.com/maps/World_Imagery.

Shorelines were digitized using geo-referenced aerial photos in ArcGIS. Digitized shoreline data was provided in part by the College of William and Mary, Virginia Institute of Marine Science, Shoreline Studies Program. The 2013 Tangier Islands Shoreline was digitized by the US Army Corps of Engineers, Norfolk District, using the ESRI Online World Imagery Basemap that was available on September 2014. The earliest map of the Tangier Islands drawn accurately enough to geo-reference within ARCGIS was created in 1850. Periodic updates of similar digitizing quality include the shorelines of 1905, 1938, 1943, 1960, 1987, 2001, 2009, and 2013 and are the subject of our analysis and basis of future predictions for the Tangier Islands. Fully-digitized shoreline data were available for 1850, 1905, 1938, 1943, 2009 and 2013, with partial data available (western shoreline) for the 1960, 1987 and 2001 (Fig. 1, Supplemental Fig. 1).

The Tangier Islands were examined for the rate of shoreline loss over time (1850–2013) using the Digital Shoreline Analysis System from USGS^21^. The overall historic rate of land loss from all three (Goose, Uppards, Tangier) Tangier Islands was determined using available shoreline data and further analyzed to assess the rate over time. We discarded transects that did not touch all shorelines, as well as transects that were not striking the shoreline at an angle close, or perpendicular, to the shore, in order to obtain accurate results (Supplemental Fig. 2). Further statistical analyses were done to determine if the rate of the western shoreline and eastern shoreline retreats differed, and if they differed between Tangier and Uppards Islands. We also conducted analyses (multiple linear regression) on the shoreline erosion data to determine, if possible, on which shorelines erosion was dominated by storm-induced erosion and/or RSLR.

**Figure 2:**
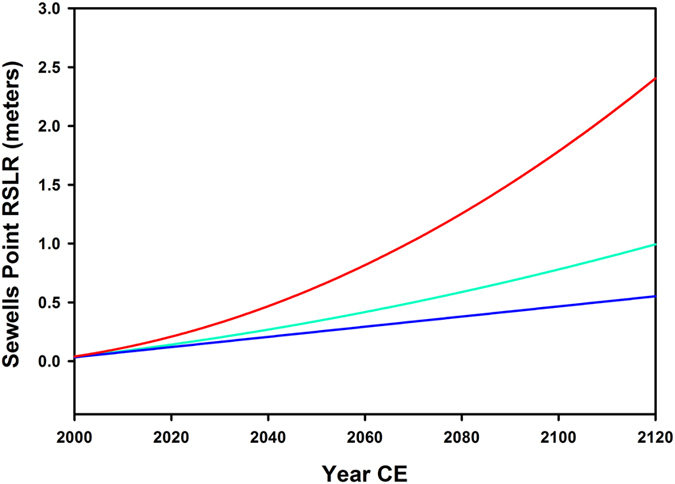
Local RSLR over time at Sewell’s Point, Virginia, used in all analyses. Blue line is the low- RSLR scenario, teal line is the mid-RSLR scenario, and red is the high-RSLR scenario.

**Figure 3:**
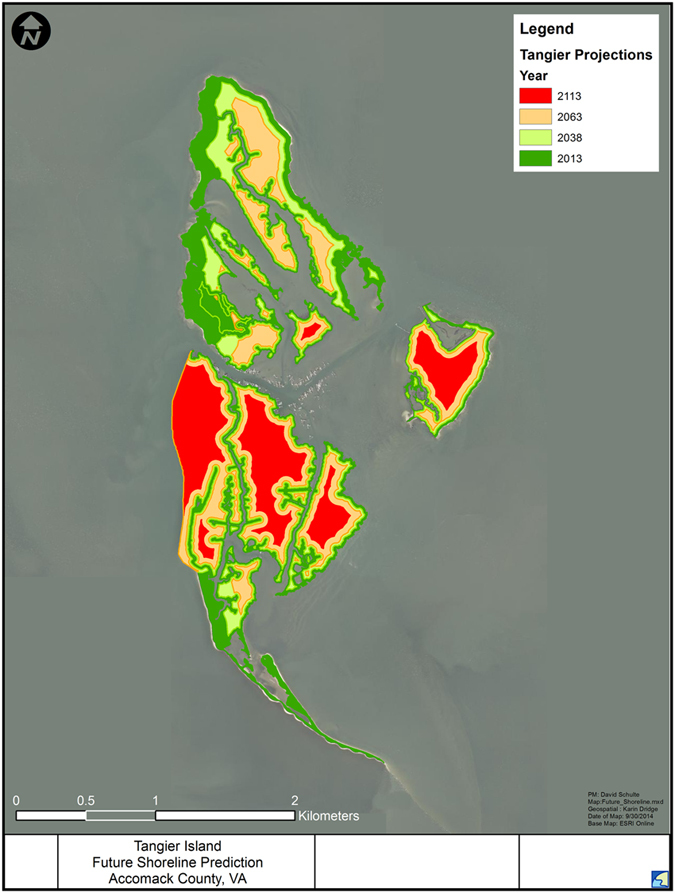
Current and projected future landmass of the Tangier Islands. Map created using ESRI ArcGIS, ArcMap 10.1. Source: Esri, DigitalGlobe, GeoEye, Earthstar Geographics, CNES/Airbus DS, USDA, AEX, Getmapping, Aerogrid, IGN, IGP, swisstopo, and the GIS User Community. For more information on this map, including the terms of use, visit: http://goto.arcgisonline.com/maps/World_Imagery.

Applying the rate of shoreline retreat calculated for the Western Shore of Uppards, Eastern shorelines of Uppards and Tangier, and internal land loss, projections were made in ARCGIS to predict the amount of land loss and island configuration in 25, 50 and 100 years from a 2013 baseline. We used the local RSLR (Sewells Point) as it is very close to the southern Chesapeake Bay mean of 4.0 mm yr^−1^ 9,22 to represent present mean sea level (PMSL). The tidal gage at Sewells Point is calibrated to NAVD88 datum. Three scenarios were chosen - low, mid, and high range - for future RSLR in Southern Chesapeake Bay (Fig. 2). These RSLR projections were then used, along with the historic rates of land loss, to project the appearance and acreage of the Tangier Islands into the future in 25, 50 and 100 years (Fig. 3).

Due to the lack of information on historic acreages of wetlands and uplands, the present study focused only on the rate of land loss of the Islands, not conversion of uplands to wetlands. However, as the current mean upland elevation of the Town (1.46 m above PMSL, average tidal range 0.37 m) is known, an estimate of the future habitability of the Town’s uplands can be estimated based on expected rates of RSLR; we did so using the projected rates of RSLR derived from data and equations at the website^22^.

We then developed a conceptual plan to address the erosion and RSLR issues at Tangier Islands. This plan is not part of any formal U.S. Army Corps of Engineers feasibility study (the decision document of any recommended Federal action plan by the U.S. Army Corps of Engineers) nor within any specific U.S. Army Corps of Engineers authorization (regulations that define specific Corps studies and/or missions), which would require initiation through a partnership between local interests and the Federal government.

## Results

**Figure 4:**
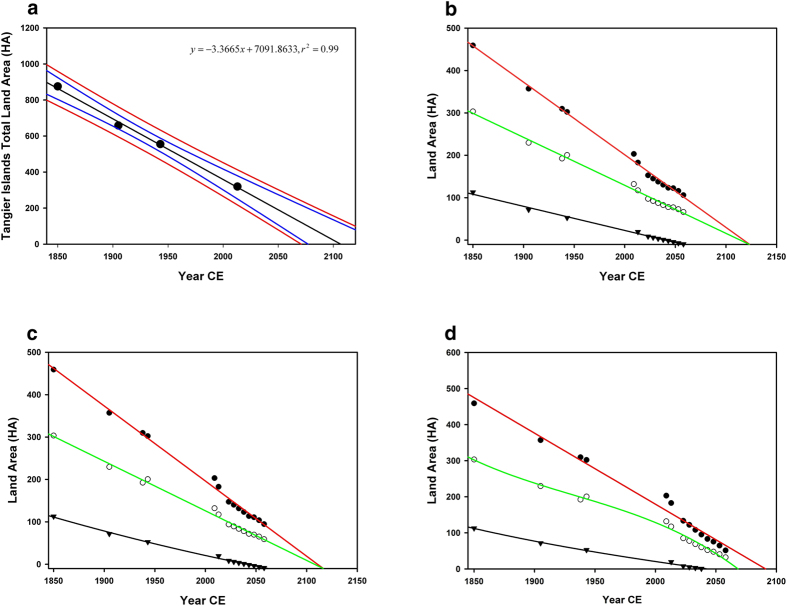
Regressions on the total Tangier Islands landmass over time. (Panel **a**) considers the total Islands landmass with the historic rate of RSLR applied, blue lines are prediction bands, red lines are the 95% CI. Remaining graphs consider the three main Islands separately (Goose = triangles, Uppards = open circles, Tangier = filled circles). (Panel **b**) considers the low-range RSLR scenario, panel c considers the mid-range RSLR scenario, and panel d considers the high-range RSLR scenario.

Land loss has been extensive since 1850, especially along the western shore (Fig. 1). As of 2013, only 33.25% (319.35 HA) of the Tangier Islands remain since 1850 (875.33 HA) giving a mean annual loss of 3.41 HA yr^−1^. Variance was highest along the western shores of the Islands, especially Uppards Island (Supplemental Fig. 2) and Tangier Island, before breakwater construction. We fit linear regressions (all parameters had p < 0.05), to the data to predict the lifespan of the island system (Fig. 4) using the historic land- loss rate projected into the future. Best fits were linear, quadratic or cubic (Supplemental Table 1). These fits suggest that, if historic rates of land loss and RSLR were to continue, the islands should be inundated by 2106, possibly as early as 2070. Goose Island, the smallest island of the three, is the first predicted to be completely submerged-before 2050 (Figs 3 and 4)-regardless of the RSLR scenario (linear, low, mid, or high) chosen. The low and mid-range RSLR scenarios predict a similar time of inundation for Uppards and Tangier by approximately 2106, with the high RSLR scenario predicting these islands will be lost by the late 2060s.

We then separated island transects of shoreline data from 1850, 1905, 1938, 1943, 1960, 1987, 2001, 2009 and 2013 into western, eastern, and interior shores using USGS Digital Shoreline Analysis System, and developed estimates of land loss over time for these shorelines. The erosion rate on the eastern shore of Tangier Island (1850–2013) was lower (86.61 m or 0.53 m yr^−1^) than that of the eastern Uppards Island shoreline (214.52 m or 1.32 m yr^−1^). In order to assess the possible impact of major storms (wind speeds from 63 to >119 km hr^-1^), we consulted historic records^23^,^24^ to identify major storms that struck the Tangier Islands (Supplemental Fig. 3) and assessed Uppards Island out of the three main islands due to its large size, similar persistence to Tangier Island and that it has not been protected by any stone breakwaters as Tangier has been. For Uppards Island eastern shoreline, RSLR is the driving factor for land losses (multiple linear regression, p > 0.291 for storms over time, p < 0.019 for RSLR over time). Major storms had significant impacts on the western shores of Uppards Island (multiple linear regression, p < 0.028 for storms, p = 0.763 for RSLR). Interior shorelines of tidal creeks have also lost significant land since 1850 (0.301 m yr^−1^); however, due to limited data we were unable to resolve the reason statistically. These estimated future rates of land loss developed from the regressions were applied when we used ARCGIS to project future land-loss scenarios.

## Discussion

Uppards is expected to be inundated at an accelerating rate compared to the semi-protected Tangier, while Goose, the smallest of the Tangier Islands, is predicted to be entirely inundated by 2038. South of the seawall, Tangier will experience significant land loss. The projection indicates it is very likely that the sand spit, which provides some protection against incoming wave energy from the south, will be lost. Tidal creeks winding through the islands will widen significantly, encroaching into the upland ridges. Most of Uppards is predicted to be inundated by 2063, reducing the protection provided to the Town of Tangier. Tangier subsides and is split into three by widening of several large tidal creeks, with continued loss of land expected to occur at all margins of the island except the western shore. By 2113, Uppards Island is predicted to be lost with the possible exception of one small area in its south-central region. A small sub-islet to the east, where there was dredged material placement activity that elevated a significant portion to uplands, is predicted to persist, though reduced in size and elevation. Tangier Island has been split into three sub-islets centered on the three upland ridges (Fig. 3).

Regarding the future of the Town of Tangier, except small portions of each ridge (<10%), the remaining upland is at a mean of +1.2 m PMSL. Based on projected rates of land loss and RSLR, the town will be uninhabitable in less than 100 years (likely by 2063) and all current uplands, with the possible exception of the three small higher areas (<10% of current uplands), will be converted to a mix of intertidal and high estuarine marsh.

### Value of Ecosystem Services Lost

Under the mid-range RSLR scenario, we expect the Tangier Islands to lose land at an exponential rate (*y* = 2*e*+16*e*^−0.016x^, *r*^2^ = 0.99). By 2063, 174.53 HA of wetlands (ecological services estimated value: 1.75 million USD yr^−1^ will be lost. By 2113, 254.41 HA will be lost, (valued at 2.54 million USD yr^−1^). The loss of Uppards will lead to the loss of approximately 150 HA of SAV by 2113, (ecological services valued at 3.0 million USD yr^−1^). Bird nesting will decline, accelerating the decline of these species^25^.

### Recommended Plan for the Tangier Islands

**Figure 5:**
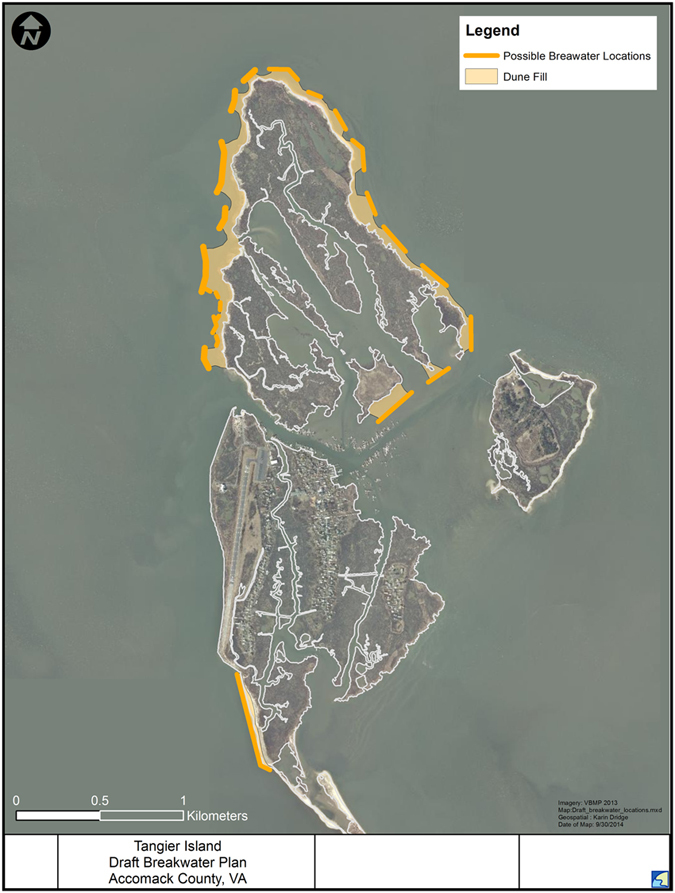
Conceptual protection/restoration plan to maintain the remaining landmass of Uppards and Tangier Island. Map created using ESRI ArcGIS, ArcMap 10.1. Source: Esri, DigitalGlobe, GeoEye, Earthstar Geographics, CNES/Airbus DS, USDA, AEX, Getmapping, Aerogrid, IGN, IGP, swisstopo, and the GIS User Community. For more information on this map, including the terms of use, visit: http://goto.arcgisonline.com/maps/World_Imagery.

The Tangier Islands have less than 100 years before they are lost to a combination of RSLR and storm surge-induced erosion. A segmented breakwater system would provide physical protection (Fig. 5). These breakwaters would be built offshore to permit construction of a sand beach/dune system along the western shore between the breakwaters and existing shoreline. The dunes would catch wind-borne sediment as well as supply inorganic sediment to the island during storms strong enough to blow some of the dune sand over the island. Along the eastern shore, breakwaters would be built.

Since 1850, five former upland ridges on Uppards and Tangier have become low-lying marsh. These would be restored using dredged sand. These ridges, restored to a height of 2–3 m above the current 2013 spring high tide, could be planted with loblolly pines (*Pinus taeda*). Pines and other woody vegetation will supply organic material to the ridges and the rest of the island, help maintain elevation, and serve as seabird nesting habitat. Spray dredging could be used to help maintain the elevation of uninhabited portions of the islands. Fertilization of the wetlands should also be considered, to increase the rate of organic material deposition where wetlands cannot be reached by spray dredging. The approximate costs of initial construction of the breakwaters, beach, dunes and ridges would be approximately 20–30 million USD. If no action is taken, significant wildlife habitat will be lost, as well as the culturally-unique Town of Tangier, the last offshore fishing community in Virginia waters of Chesapeake Bay.

## Conclusions

The available data and our analyses have shown that the Tangier Islands have lost the majority of their landmass since first accurately mapped in 1850, due to a combination of wave-induced erosion and SLR. Our future projection, which assessed a more conservative mid-range scenario, predicts the Islands will lose of the majority of their remaining landmass and that the Town of Tangier will likely have to be abandoned in less than 50 years. Significant ecological services worth millions of USD yr^−1^ will also be lost. If the high SLR scenario occurs, land losses will occur much more rapidly and the Town will have to be abandoned even sooner. A recent study^26^ has indicated that a higher SLR scenario is becoming more likely as humanity fails to take effective action to reduce carbon emissions. The US Army Corps of Engineers recognizes that climate change is upon us and that adaptation to climate change is “not optional”^27^. The Tangier Islands and the Town are running out of time, and if no action is taken, the citizens of Tangier may become among the first climate change refugees in the continental USA.

## Additional Information

**How to cite this article**: Schulte, D. M. *et al.* Climate Change and the Evolution and Fate of the Tangier Islands of Chesapeake Bay, USA. *Sci. Rep.* **5**, 17890; doi: 10.1038/srep17890 (2015).

## Supplementary Material

Supplementary Information
