# Supplementary material for: Climate Change and the Evolution and Fate of the Tangier Islands of Chesapeake Bay, USA
Source: Sci Rep. 2015 Dec 10;5:17890. doi: 10.1038/srep17890 (PMC4675185; doi:10.1038/srep17890)
Supplement: Supplementary Information [file srep17890-s1.pdf]

Title: Climate Change and the Evolution and Fate of the Tangier Islands of Chesapeake Bay, USA

Authors: corresponding: David M. Schulte, contributing: Karin M. Dridge, Mark H. Hudgins

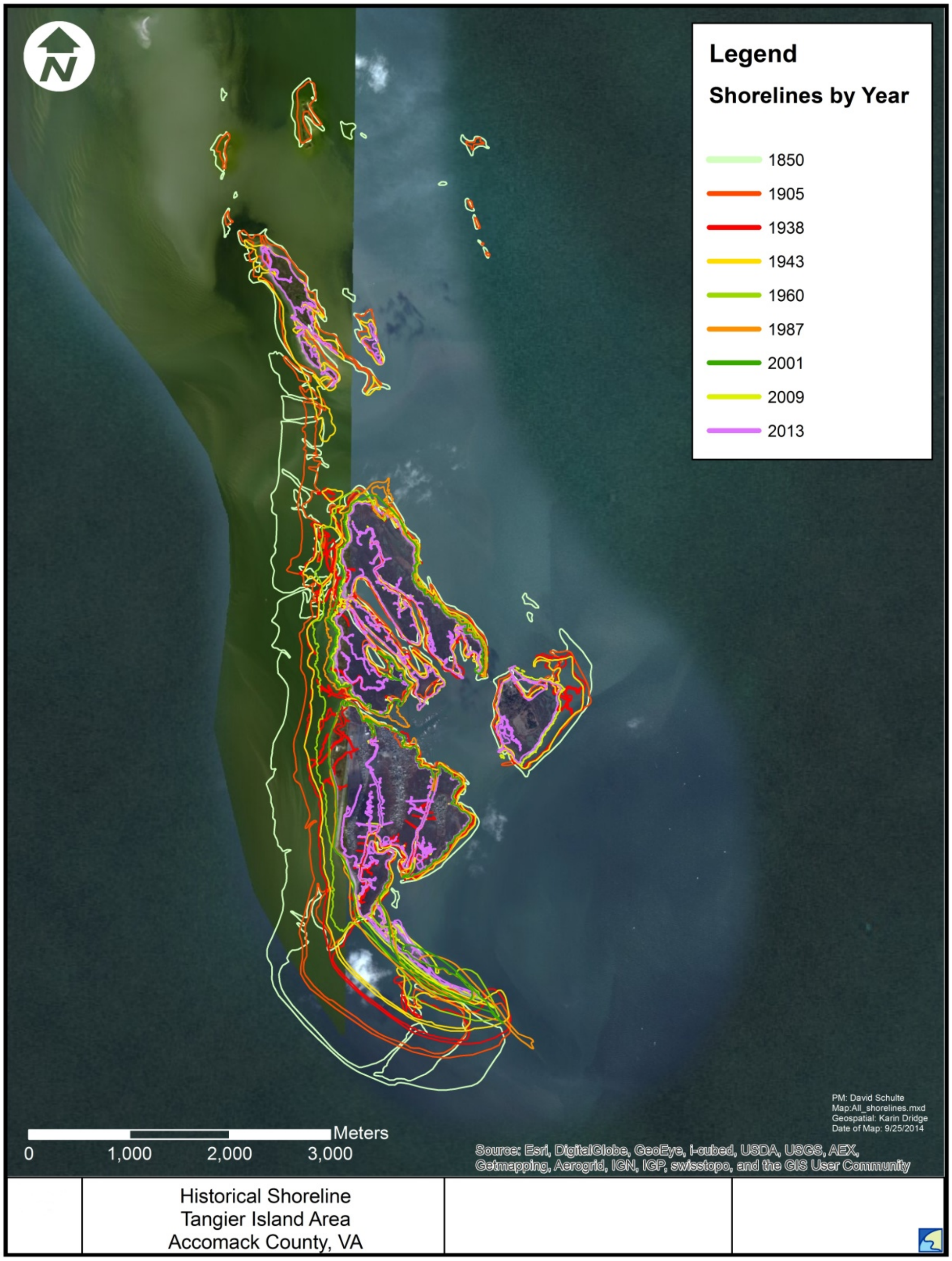

Supplementary Figure 1. Historic shorelines over time of the Tangier Islands, Virginia, Chesapeake Bay USA. Source: Esri, DigitalGlobe, GeoEye, Earthstar Geographics, CNES/Airbus DS, USDA, AEX,

Getmapping, Aerogrid, IGN, IGP, swisstopo, and the GIS User Community. For more information on this map, including the terms of use, visit: http://goto.arcgisonline.com/maps/World_Imagery.

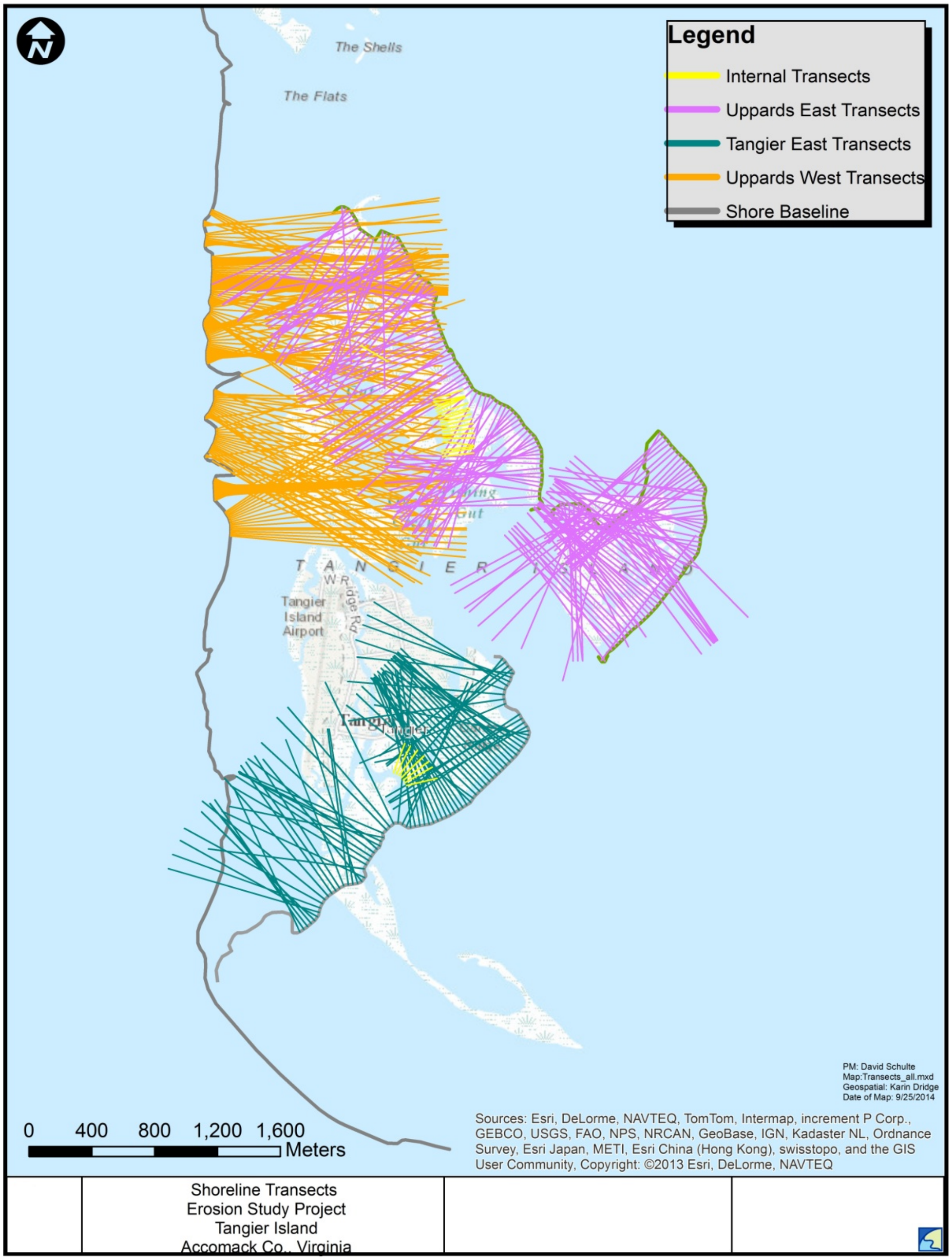

Supplementary Figure 2. Shoreline transects used to determine historic land shoreline loss rates over time. Map created using ESRI ArcGIS, ArcMap 10.1.

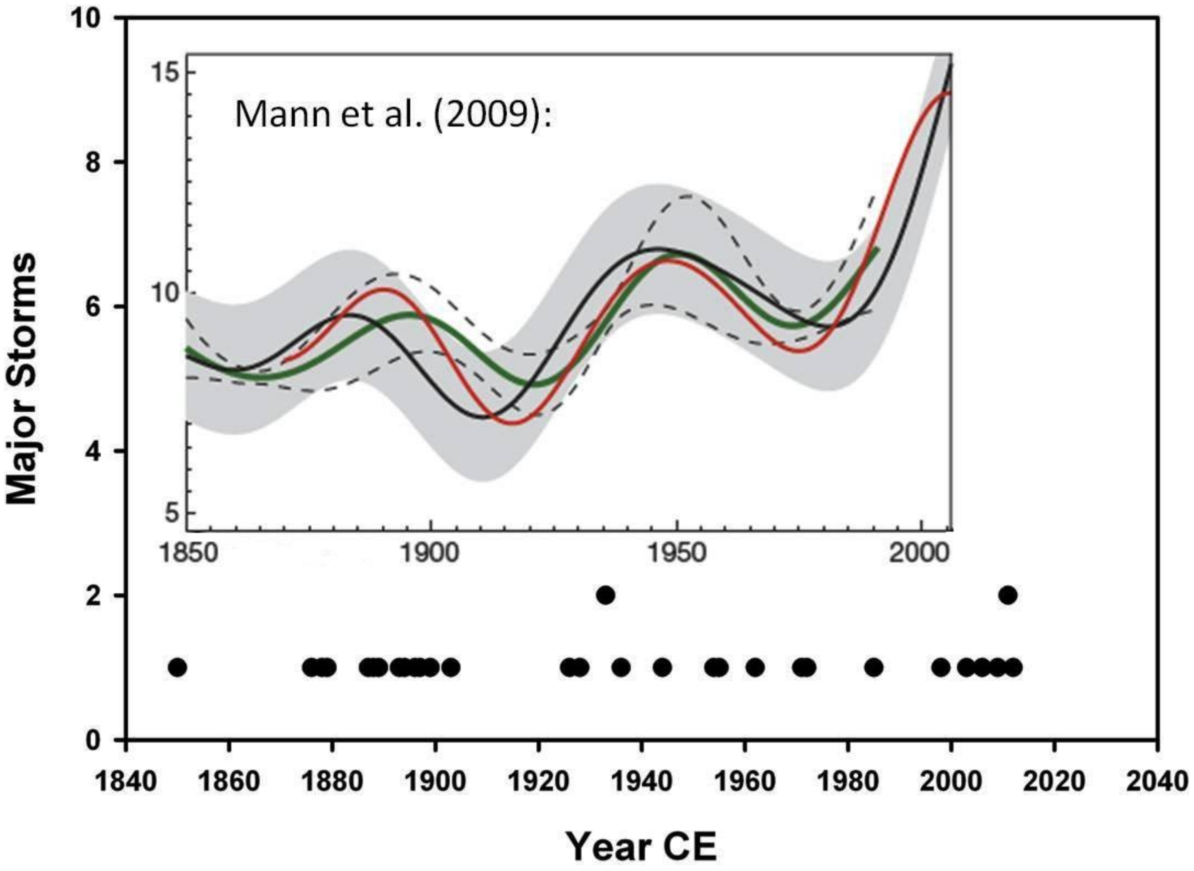

Supplementary Figure 3. Major storms in the local region of the Tangier Islands, indicated as black circles. They follow a general pattern observed in the North Atlantic by Mann et al. 2009.

SF2 Reference:

Mann, M. E., Woodruff, J. D., Donnelly, J. P., & Zhang, Z. Atlantic hurricanes and climate over the past 1,500 years. *Nature*. **460**, 880-883 (2009).

SLR Scenario	Island	Regression	Best-Fit Equation	r^2	p
Low	Goose	$f = y + ax$	$y = 1160.1508 - 0.5687x$	0.99	y < 0.0001, a < 0.0001
Low	Uppards	$f = y + ax$	$y = 2394.3311 - 1.1326x$	0.99	y < 0.0001, a < 0.0001
Low	Taniger	$f = y + ax$	$y = 3627.3598 - 1.7131x$	0.99	y < 0.0001, a < 0.0001
Mid	Goose	$f = y + ax + bx^2$	$y = 3152.8223 - 2.6069x + 0.0005x^2$	0.99	y = 0.0035, a = 0.0114, b = 0.035
Mid	Uppards	$f = y + ax$	$y = 2465.3982 - 1.1697x$	0.99	y < 0.0001, a < 0.0001
Mid	Taniger	$f = y + ax$	$y = 3740.2302 - 1.772x$	0.99	y < 0.0001, a < 0.0001
High	Goose	$f = y + ax + bx^2$	$y = 3972.5593 - 3.4514x + 0.0007x^2$	0.99	y = 0.0014, a = 0.0039, b = 0.0105
High	Uppards	$f = y + ax + bx^2 + cx^3$	$y = 199766.2418 - 307.8088x - 0.1588x^2 - 2.786e^{-.005}x^3$	0.99	y = 0.0432, a = 0.0428, b = 0.0415, c=0.0397
High	Taniger	$f = y + ax$	$y = 4120.2245 - 1.9704x$	0.98	y < 0.0001, a < 0.0001

Supplementary Table 1. Best-fit regression equations used for low, mid and high SLR scenarios for Goose, Uppards and Tangier Islands.
